# Different neural mechanisms for nonsalient trained stimuli and physically salient stimuli in visual processing

**DOI:** 10.1002/pchj.718

**Published:** 2023-12-27

**Authors:** Zile Wang, Qi Zhang, Yuxiang Hao, Shuangxing Xu

**Affiliations:** ^1^ School of Education and Psychology Minnan Normal University Zhangzhou China; ^2^ Institute of Applied Psychology Minnan Normal University Zhangzhou China; ^3^ Fujian Province Key Laboratory of Applied Cognition and Personality Zhangzhou China

**Keywords:** EEG, neural mechanism, nonsalient trained stimuli, physically salient stimuli

## Abstract

Previous studies have shown that nonsalient trained stimuli could capture attention and would be actively suppressed when served as distractors. However, it was unclear whether nonsalient trained stimuli and physically salient stimuli operate through the same attentional neural mechanism. In the current study, we investigated this question by recording event‐related potentials (ERPs) of searching for the two stimuli separately after matching the difficulty. The present results provided additional evidence for the function of the suppression in that it may terminate a shift of attention. For the N1 component, the nonsalient trained stimuli had a shorter latency and larger amplitude than the physically salient stimuli whether presented as targets or distractors. It indicated that the nonsalient trained stimuli had an earlier sensory processing and greater visual attention orienting. The N2 posterior‐contralateral (N2pc) amplitude of the physically salient target was larger than the nonsalient trained target. This suggested that physically salient stimuli had a stronger ability to capture attention. However, when they presented as distractors, only the nonsalient trained stimuli could elicit the P_D_ component. Therefore, active suppression of the physically salient stimuli was more difficult than the nonsalient trained stimulus with the same difficulty. For the P3 component, the amplitude of the physically salient stimuli was larger than that of the nonsalient trained stimuli, both as targets and distractors, which indicated that the top‐down controlled process of outcome evaluation for the salient triangle was stronger. Overall, these results suggested that they were processed via different neural mechanisms in the early sensory processing, attentional selection, active suppression, and the outcome‐evaluation process.

The attentional capture of physically salient stimuli has been extensively studied in the past few decades (Burra & Kerzel, 2014; Gaspelin et al., 2017; Geng & Di Quattro, 2010; Kerzel & Schönhammer, 2013; Kiss et al., 2012; Serences et al., 2005; Turatto & Galfano, 2000). Physically salient stimuli are those in which at least one feature (e.g., color, shape, orientation, luminance) is different from the other stimuli during visual search (Turatto & Galfano, 2000, 2001). The capture is automatic, inevitable, and attention is always first distributed on the most salient stimulus, irrespective of the intentions or goals of the observer (Theeuwes, 1992, 1994; Yantis & Jonides, 1984). Some researchers have found that when physically salient stimuli are presented as distractors, there is a suppression mechanism to prevent the capture attention of salient distractors (Gaspar & McDonald, 2014; Gaspelin et al., 2017; Sawaki & Luck, 2010). There are two different functions of attentional suppression to explain this mechanism. The signal suppression hypothesis consider that a priority signal of “attention to me” is generated automatically by these salient distractors, and the attention deployment toward these salient distractors can actually be avoided (Sawaki & Luck, 2010, 2011). This hypothesis reflects that the attentional suppression may serve to prevent a shift of attention. In this case, the N2 posterior‐contralateral (N2pc) component disappear without subsequent P_D_ component. Another function of the attentional suppression is that it may terminate a shift of attention. In other words, the same suppression mechanisms that used to prevent attention from shifting to distractors may also be employed to terminate attention after it has already been focused on the relevant object (Sawaki et al., 2012). If the shift of attention is terminated by the active suppression mechanism, the N2pc component should be followed by the P_D_ component.

The impact of physically salient stimulus on behavioral performance is considerably similar to nonsalient trained stimuli. In fact, most studies of attentional capture have focused on how salient stimuli capture attention and whether it can be actively suppressed. Some researchers have revealed that nonsalient stimuli with sufficient training could also capture attention automatically (Kyllingsbæk et al., 2001, 2014; Lin et al., 2017; Qu et al., 2017). Qu et al. (2017) studied attentional capture of nonsalient stimuli (shapes) and found that the nonsalient trained shapes could capture attention and elicit N2pc components. Hu et al. (2018) used a similar experimental paradigm and found that nonsalient shapes not only elicited N2pc components but also P_D_ components when presented as distractors after visual search training. It showed that the process of active suppression applied not only to salient features but also physically nonsalient shapes.

In general, both physically salient and nonsalient trained stimuli share many important characteristics. For nonsalient stimuli, some researchers have argued that higher‐level brain areas (e.g., frontal and parietal cortex) related to attentional control or decision‐making play an essential role in visual search training (Ahissar & Hochstein, 2004; Dosher & Lu, 1998; Zhang et al., 2010). For physically salient stimuli, the neuroimaging finding has highlighted the influence of the frontal cortex in mitigating salience‐driven distraction (Aron, 2011; Bari & Robbins, 2013; Leber, 2010; McNab & Klingberg, 2008). Furthermore, some electrophysiological evidences have shown that both nonsalient trained stimuli and physically salient stimuli could elicit the N2pc component when presented as targets and P_D_ components when presented as distractors (Burra & Kerzel, 2014; Hu et al., 2018; Luck et al., 2021; Qi et al., 2013; Qu et al., 2017; Sawaki & Luck, 2010). These results seem to suggest that there are similarities at some level in their underlying neural mechanisms. However, in previous studies, the mechanism of different cognitive stages represented by different ERP components has not been explored and few studies have compared the cognitive mechanisms of nonsalient trained and salient stimuli at the same difficulty of search task.

In the current study, each subject participated in the behavioral training and test phase, the matching phase and the EEG test phase over a period of 4 days (Figure 1A). In the behavioral training and test phase, subjects performed the visual search training task for 3 days. Before and after the visual search training, the subjects conducted the pretest and posttest (the pretest on the first day and the posttest on the third day). In addition, on the third day, we matched the task difficulty of two stimuli after the posttest, thereby excluding the effect of task difficulty on the results. On the fourth day, we recorded the EEG data when the subjects were performing the central rapid serial visual presentation (RSVP) task and visual search tasks. In the visual search tasks, we analyzed N1, N2pc, P_D_, and P3 components, which represented the early sensory processing, attentional selection, active suppression, and the outcome evaluation process, respectively, to explore the difference of neural mechanisms for nonsalient trained stimuli and physically salient stimuli in visual processing. We mainly analyzed the latency and amplitude of EEG components elicited by nonsalient trained stimuli and physically salient stimuli when presented as targets and distractors. We speculated that the latency and amplitude of the N1, N2pc, P_D_, and P3 components elicited by the two stimuli were different. However, nonsalient trained stimuli and physically salient stimuli were presented as targets or distractors in visual search tasks. In the central RSVP task, the trained and salient shape was presented peripherally and was neither spatially nor featurally relevant to the task goal (the task was to identify the central blue letter). Therefore, we excluded the impact of top‐down attentional control of the two stimuli and provide clear and novel evidence supporting the existence of a purely bottom‐up capture of attention by the geometric shape. In the central RSVP task, we considered the situation that these two stimuli were task‐irrelevant and presented outside the focus of top‐down spatial attention to study the involuntary, bottom‐up capture of attention. In addition, we also tested which of the two different functions of attentional suppression previously mentioned might underlie the suppression process of task‐irrelevant stimuli and distractors. If we observed that N2pc gradually weakens until it disappears, it would suggest that suppression may serve to prevent a shift of attention. Of course, if we observed the P_D_ component after N2pc, it may have terminated a shift of attention.

**FIGURE 1:**
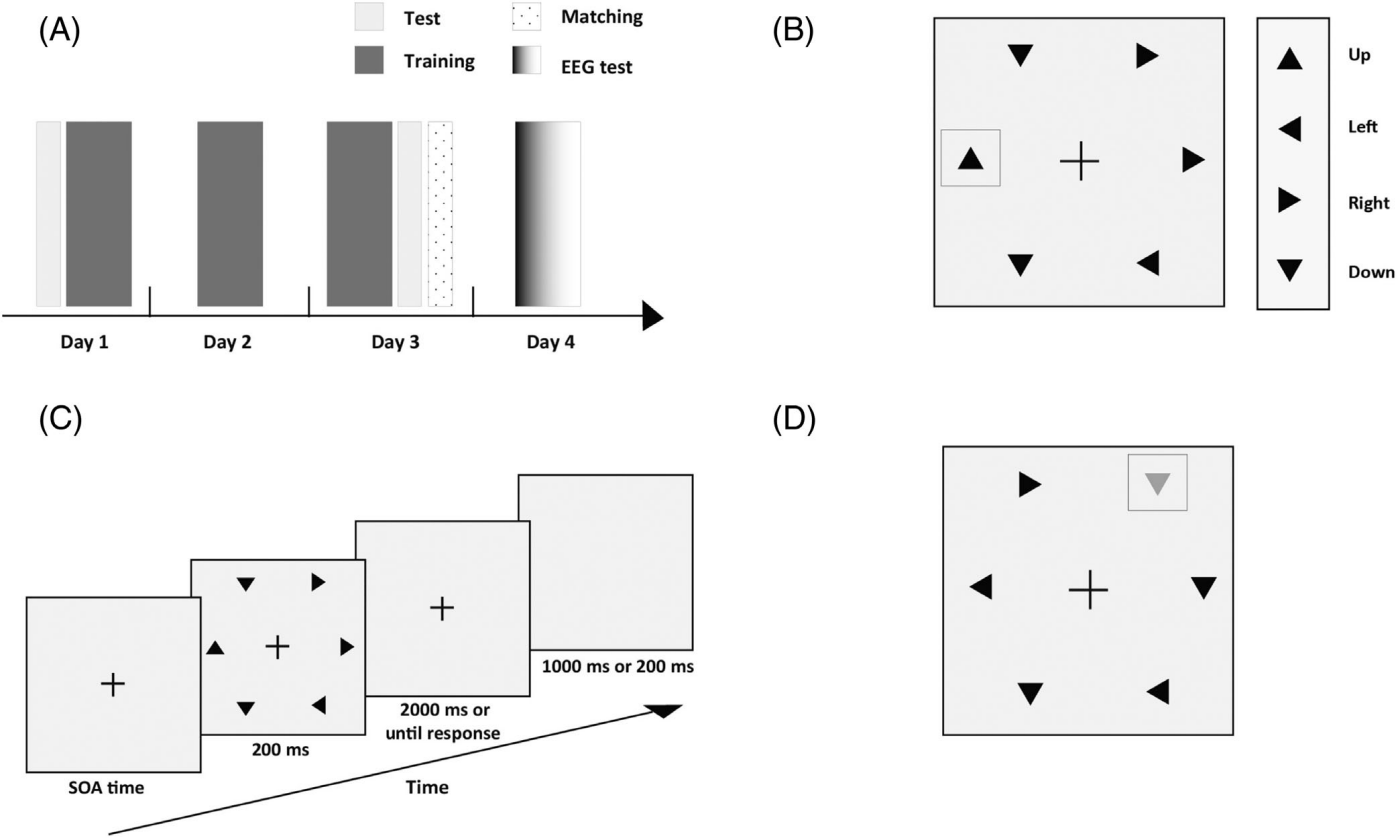
(A) The procedure in the whole experiment. (B) Examples of stimuli used in visual search. (C) The sequence of displays in a typical trial of visual search task. There was a blank screen for 1000 ms in the pretest and posttest, the matching phase, and the search tasks in the EEG test phase. The blank screen was 200 ms in the behavioral training phase. (D) Examples of salient stimuli used in the matching phase and visual search tasks.

## METHODS

### Subjects

According to priori power analysis, at least 14 subjects were required to replicate this effect in repeated‐measure analysis of variance (ANOVA; $\eta_{p}^{2}$ = .125, power = 0.9). The effect size ($\eta_{p}^{2}$) was based on the study of visual search training from Schuster et al. (2013). Therefore, we recruited 21 healthy young adults who participated as paid volunteers in this study (mean age ± *SD*: 20.9 ± 2.8 years; 12 female), and all the subjects were college students. All had normal or corrected‐to‐normal vision, and were naive to the purpose of the study. Written informed consent was obtained from all subjects before the experiment. In the analysis of behavioral training and test phase, the results of all subjects were included. For the EEG test phase, one subject was rejected from the further analysis in the visual search tasks because of misremembering the target. In the central RSVP task, two subjects were excluded from further analysis due to excessive EEG artifacts and one subject (the same subject as in the visual search task) was rejected from the further analysis because of misremembering the target. Therefore, 20 subjects (mean age ± *SD*: 21.1 ± 2.8 years; 11 female) were included for further analysis in visual search tasks, and 18 subjects (mean age ± *SD*: 21 ± 2.8 years; 10 female) were included for further analysis in the central RSVP task. This study was approved by the Psychology and Education Research Ethics Committee of Minnan Normal University.

### Apparatus and stimuli

The Psychtoolbox 3.0 (Brainard, 1997; Pelli, 1997) in the MATLAB (MathWorks) environment was used to present the experimental stimuli. The subjects were seated in a dimly lit room, 57 cm in front of the screen (width 51 cm, spatial resolution 1680 × 1050 pixels, refresh rate 60 Hz), and head supported by a chin rest. The stimuli array consisted of six triangles (RGB: 0, 0, 0) presented 7° from the center of screen (Figure 1B). The edge length of all triangles was 1°. All stimuli were presented on a gray background (RGB: 127, 127, 127).

### Procedure

#### Behavioral training and test phase

There were four different orientations of the triangle (Up, Down, Left, or Right). For each subject, one of them was selected randomly as the target (e.g., Up), and the other three triangles in different orientations were presented randomly as distractors with an equal overall probability. In this case, there would be two Down triangles, two Left triangles, and two Right triangles in the stimulus display when the target was absent (50% of trials). One of the orientations among the distractors (Right, Down, or Left triangles) was replaced with the Up triangle (target) when the target was present (50% of trials), and the positions of six triangles were constant during the experimental task. In the trained search task, we took the triangles that the subjects trained to search as the trained triangle (e.g., Up triangle). In the behavioral training stage, the Trained triangle would receive 3 days of training with 1,260 trials each day. The experiment began with a cross lasting 600–1000 ms. Then, a search display was presented for 200 ms (Figure 1C), and a central fixation cross did not appear until a response or 2000 ms had passed after its onset. The response was recorded only before the fixation cross disappeared. The task of the subjects was to determine whether or not the target was present as fast as possible. Subjects pressed the left arrow button when the target appeared and pressed the right arrow button when the target was absent. Then, a blank screen was presented for 1000 ms in the pretest and posttest and for 200 ms in the behavioral training stage after response. Subjects were asked to stare at the central cross during the search.

To explore the specific learning effects, we tested two kinds of search tasks in the pretest and posttest. Each task included 120 trials. One search task had the same target as the trained search task (searching for the Trained triangle). For the other task, there was an untrained search task in which subjects were asked to search for the triangle that had been used as distractors (Down, Left, or Right). We called the target in the untrained task the “Untrained triangle” (e.g., Right, called Untrained triangle).

The Trained and Untrained triangles were selected randomly (the Trained and Untrained triangles were held constant within subjects and randomized between subjects). Before the search task, the subjects were told which triangle to search for (e.g., the subjects were told: “The target you are going to search for is the Up triangle, if you see it appear, you press the left button; if not, press the right button”).

#### Matching phase

A matching phase was required to ensure that the difficulty of searching for the nonsalient trained triangle was the same as that for the physically salient triangle before EEG test. In order to be comparable, we need to use uniform luminance values in different conditions. If we selected the luminance value when the salient stimulus was present as a distractor, the salient stimulus was not presented as the only distractor (there were other oriented triangles as distractors). In this case, the task difficulty was not purely due to the salient stimulus, thus making it even more unreliable. In this way, we selected the luminance value when searching for the salient stimulus for matching, because the salient stimulus was unique when it presented as a target. After the posttest, subjects completed another block of searching task, and the target of this task was the salient triangle (Figure 1D). There were six conditions for the salient triangle: (the RGB for six conditions: condition 1 [RGB: 40, 40, 40]; condition 2 [RGB: 50, 50, 50]; condition 3 [RGB: 60, 60, 60]; condition 4 [RGB: 70, 70, 70]; condition 5 [RGB: 80, 80, 80]; condition 6 [RGB: 90, 90, 90]). The saliency was determined with respect to the other search elements rather than the background. The triangles of other orientations were presented as distractors except the Trained triangle.

There were 216 trials in the whole matching phase. The typical sequence of the trial in the matching phase was similar to the pretest and posttest (Figure 1C), except that the target was replaced by the salient triangle (Figure 1D).

The mean accuracy (p') of searching for the Trained triangle was calculated for each subject in the posttest (mean ± *SD*: 0.85 ± 0.10). For each subject, we calculated the accuracies of searching for the salient stimulus under different conditions. The curve was gained by the following Sigmoid curve fitting.

$$y=\text{base}+\frac{\max}{1+\exp\left(\frac{\text{xhalf}-x}{\text{rate}}\right)}.$$

In the formula above, the luminance value was *x*, and accuracy was *y*. We calculated the luminance value of each subject according to the obtained curve and the average accuracy of the posttest as y after Sigmoid curve fitting (mean = 75.06; *SD* = 8.87). This luminance value was used for the salient stimulus in the EEG test phase.

#### EEG test phase

**FIGURE 2:**
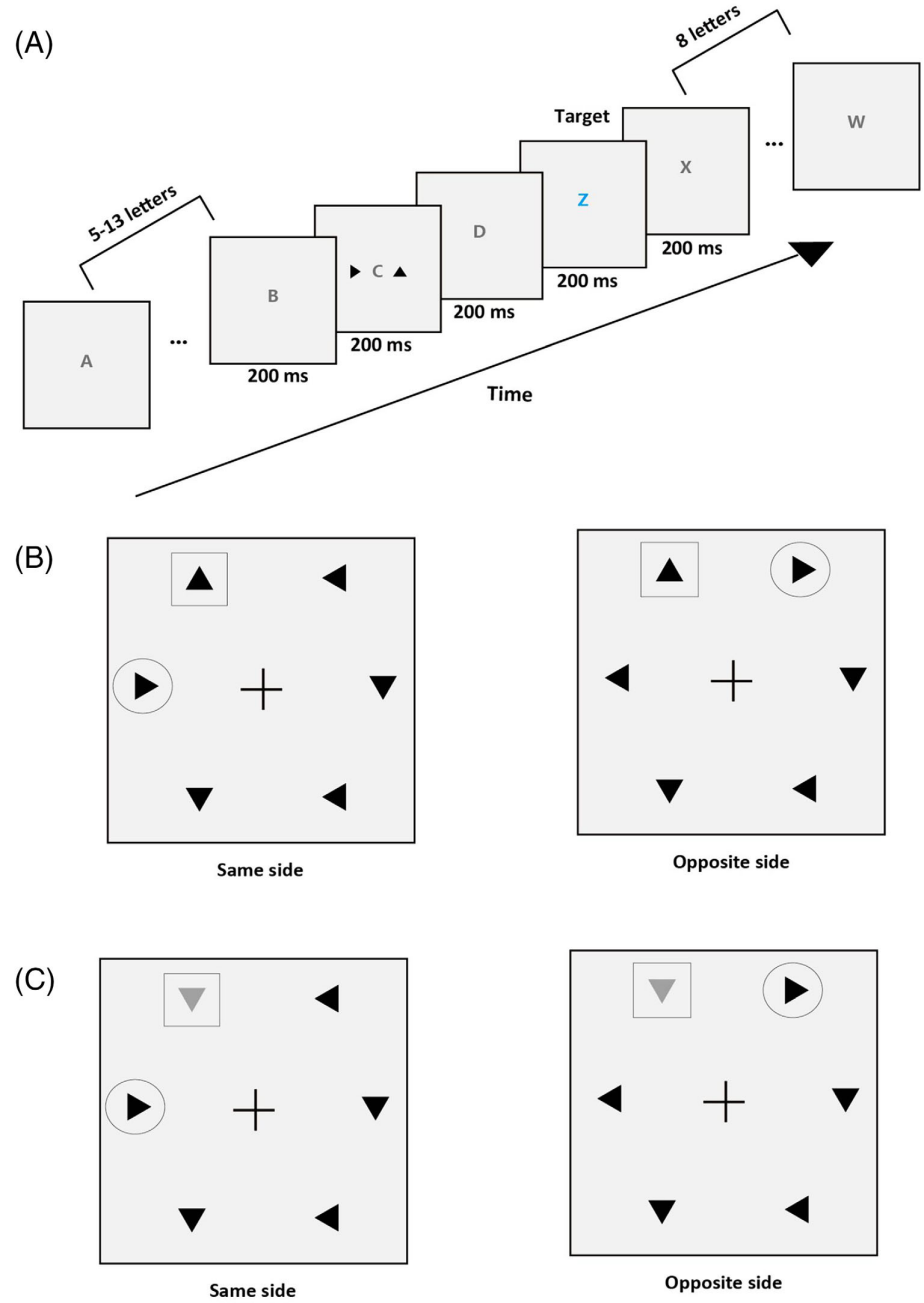
(A) The sequence of displays in a typical trial of the central RSVP task. (B) Examples of nonsalient stimulus used in visual search tasks. The Up triangle (marked with the square, not shown during experiments) was presented as the Trained triangle and the Right triangle (marked circular, not shown during experiments) was presented as the Untrained triangle. The “same side” meant that the spatial location relationship of the Trained triangle and Untrained triangle were on the same side. The “opposite side” meant that the spatial location relationship of the Trained triangle and Untrained triangle were on the opposite side. (C) Examples of salient stimulus used in visual search tasks. “Same side” meant that the spatial location relationship of the Salient triangle (marked with the square, not shown during experiments) and Untrained triangle (marked circular, not shown during experiments) were on the same side. “Opposite side” meant that the spatial location relationship of the Salient triangle and Untrained triangle were on the opposite side.

In the EEG test phase, subjects performed an RSVP task before completing four visual search tasks. In this RSVP task, every trial included a sequence of rapidly flashing letters in the center of the display (Figure 2A). All letters were selected randomly from the 26 letters of the alphabet. The blue and white letters were displayed at the center of screen (RGB for blue: [0, 0, 255]; RGB for white: [255, 255, 255]). The height in degrees of visual angle for all letters was 1.2° and the font of the letters was Times New Roman. Each letter was presented for 200 ms and there was no interval between letters. The task of the subjects was to identify the blue letters by pressing the corresponding keys (press the left arrow key when *Z* appears, and press the right arrow key when *M* appears). A pair of triangles (the same size as the triangles in the visual search) was presented with two letters before blue target letters. The triangle pair was presented for 200 ms. There were three types of triangle pairs in the central RSVP task (trained‐distractor triangle, salient‐distractor triangle, and untrained‐distractor triangle pairs). Among the 18 subjects for further analysis in the central RSVP task, 13 subjects completed the task for a total of 600 trials. For the other five subjects, they completed with a total of 400 trials only for the first and second triangle pairs. There were 200 trials for each triangle pair condition. The number of central letters presented before the peripheral triangle pair was randomized from 5 to 13, and the number of letters after the peripheral triangle pair was fixed at 10.

The sequence of displays in a typical trial of visual search tasks was similar to pretest and posttest. The four visual search tasks included searching for the Trained triangle (Untrained triangle as one of the distractors), Untrained triangle (Trained triangle as one of the distractors), Salient triangle (Untrained triangle as one of the distractors), and Untrained triangle (Salient triangle as one of the distractors). These four search tasks are denoted by T_UnT, UnT_T, S_UnT, and UnT_S, respectively, in the following. The four tasks were performed randomly, each search task consisted of 432 trials, each block of 108 trials and the target was presented in half of the trials. Subjects were given a rest of 1–3 min between blocks. After each task was completed, the subjects took a long rest (7–10 min) to prevent fatigue.

In the task of searching for T_UnT and UnT_T, the Trained triangle and Untrained triangle we used were the same as that we tested before (e.g., Up, called Trained triangle; Right, called Untrained triangle). The Trained triangle and Untrained triangle were presented at the same or opposite side in the stimulus array (Figure 2B). In this case, in order to calculate the ERP components elicited by the Trained triangle (Up) and Untrained triangle (Right), only one Trained triangle (Up) and one Untrained triangle (Right) could appear in the stimulus array. Therefore, when the target was the Trained triangle (Up) in the target‐present trials (50% of trials), there would be one Up triangle, one Right triangle, two Down triangles, and two Left triangles in the stimulus array. In the target‐absent trials (50% of trials), the target (Up) would be replaced with a Down or Left triangle. The ERPs were calculated by subtracting voltages ipsilateral to the stimulus of interest from electrodes contralateral to the same stimulus in the same set of trials.

In the task of searching for S_UnT and UnT_S, the Untrained triangle was the same as the behavioral training and test phase, and the Trained triangle was not presented in either task. We randomly selected one of the other two orientations of the triangle except the Trained and Untrained triangles as the Salient triangle. For example, if the Right triangle was presented as the Untrained triangle, in the target‐present trials (50% of trials), there was only one Untrained triangle (Right), and the Salient triangle was selected randomly from the Down triangle and Left triangle. The remaining four triangles were two Down triangles and two Left triangles. The spatial location relationship of the Salient triangle and Untrained triangle is presented in Figure 2C. The positions were selected randomly for the Salient and Untrained triangle with half of the trials presented on the same side and half on the opposite. In the target‐absent trials (50% of trials), when the search task was to search for the Salient triangle, the Salient triangle was replaced by one of the distractors that was not the Untrained triangle (Down triangle or Left triangle); when the search task was to search for the Untrained triangle, the Untrained triangle was replaced by one of the distractors that was not the Salient triangle (Down triangle or Left triangle).

### Analysis

The search tasks in both the behavioral training phase and the EEG testing phase used the following formula to calculate the target detection accuracies:

$$p^{\prime }=\frac{p-\mathrm{fp}}{1-\mathrm{fp}},$$

where *p* and *fp* refer to the proportions of positive and false‐positive responses, respectively (Hu et al., 2018; Qu et al., 2017). Only correct trials were included in the analyses of reaction times (RTs) when the target was presented. According to Bayesian hypothesis testing (Wasserstein & Lazar, 2016; Wagenmakers et al., 2018; Wetzels et al., 2011), we calculated the Bayes factor (*BF*_10_) in order to assess the strength of the evidence for the alternative hypothesis (H1) relative to the null hypothesis (H0).

### EEG acquisition and processing

The EEG signals were recorded using a 64‐channel amplifier (ANT Neuro EEGO) mounted in a cap using a 10/20 montage. CPz served as the online reference, and GND served as the ground electrode. The EEG signals were digitized online at a sampling rate of 1000 Hz. Electrode impedances were kept below 20 kΩ.

All off‐line analyses were conducted using the EEGLAB Toolbox (Delorme & Makeig, 2004) and MATLAB (Math Works). The raw EEG was re‐referenced to the average of the left and right mastoids off‐line. The EEG signals were bandpass filtered from 0.1 to 40 Hz, and epochs were extracted that included 100 ms of pre‐stimulus and 600 ms of post‐stimulus EEG. We performed a series of preprocessing steps to remove eye movements and artifacts: (1) Trials were automatically excluded from EEG analyses if the EEG range exceeded ±50 μV. (2) To make sure that the EEG would not be contaminated by eye movements and eye blinks, we ran an independent component analysis and removed ICA components representing blinks or horizontal eye movements. (3) Sporadic artifacts, such as electrode drifts, were manually rejected. Baseline corrected relative to the 100 ms before stimulus period. Overall, an average of 6.59% of trials (range = 3.60%–9.32%) in the central RSVP task and an average of 2.03% of trials (range = 0.58%–3.48%) in the search tasks were rejected due to these artifacts.

## RESULTS

### Behavioral results

#### Behavioral training and test phase

**FIGURE 3:**
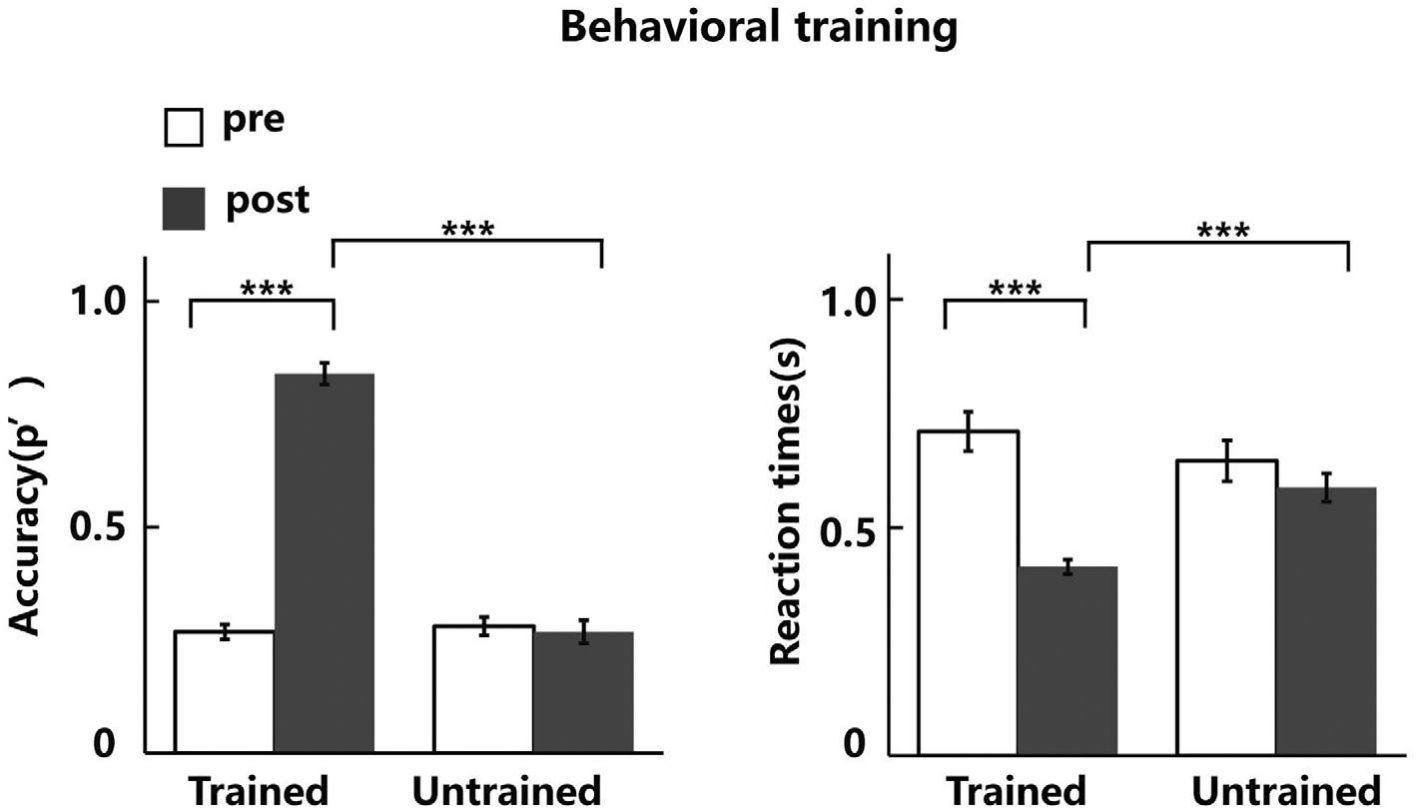
The accuracy (p') and RTs of behavioral training phase in pretest and posttest *** *p* < .001.

For accuracy (p') and reaction times (RTs), a two‐way repeated‐measures ANOVA was conducted with the factors of target types (Trained vs. Untrained triangle) and test stage (before training vs. after training) (Figure 3). The accuracy results showed significant effects on target types (*F*(1, 20) = 200.77, *p* < .001, $\eta_{p}^{2}$ = .91), test stage (*F*(1, 20) = 183.74, *p* < .001, $\eta_{p}^{2}$ = .90), and their interaction (*F*(1, 20) = 417.15, *p* < .001, $\eta_{p}^{2}$ = .95). Further analysis simple‐effects analysis (Bonferroni‐corrected) revealed that the trained triangle exhibited significant improvement in the accuracy of the search task after training (Δ −0.57 ± 0.12, *t* (20) = −22.29, *p* < .001, Cohen's *d* = 4.99, 95% CI [−0.62, −0.52]). However, we did not find a significant effect on the Untrained triangle before training and after training (Δ 0.01 ± 0.11, *t* (20) = 0.48, *p* = .637, Cohen's *d* = 0.11, *BF*_10_ = 0.25, 95% CI [−0.04, 0.06]). There was no significant difference in accuracy between the target types before training (Δ −0.01 ± 0.11, *t* (20) = −0.50, *p* = .620, Cohen's *d* = 0.11, *BF*_10_ = 0.26, 95% CI [−0.06, 0.04]). After training, there was a significant difference between the accuracy of the two target types (Δ 0.57 ± 0.11, *t* (20) = 23.62, *p* < .001, Cohen's *d* = 5.28, 95% CI [0.52, 0.62]). For RTs, the results indicated a significant effect on target types (*F*(1, 20) = 8.37, *p* = .009, $\eta_{p}^{2}$ = .30), test stage (*F*(1, 20) = 25.49, *p* < .001, $\eta_{p}^{2}$ = .56), and their interaction (*F*(1, 20) = 33.58, *p* < .001, $\eta_{p}^{2}$ = .63). Further simple‐effects analysis (Bonferroni‐corrected) revealed that the Trained triangle exhibited significant improvement in the search task after training (Δ 30 ± 17 ms, *t* (20) = 8.20, *p* < .001, Cohen's *d* = 1.83, 95% CI [0.22, 0.07]). However, the Untrained triangle showed no significant change (Δ 6 ± 21 ms, *t* (20) = 1.32, *p* = .203, Cohen's *d* = 0.29, *BF*_10_ = 0.47, 95% CI [−0.03, 0.15]). There was no significant difference in RTs between the target type before training (Δ 6 ± 14 ms, *t* (20) = 2.05, *p* = .053, Cohen's *d* = 0.46, *BF*_10_ = 1.36, 95% CI [−0.00, 0.12]). After training, there was a significant difference between the two target types in RTs (Δ −17 ± 11 ms, *t* (20) = −7.10, *p* < .001, Cohen's *d* = 1.59, 95% CI [−0.23, −0.12]). These results indicated that the RTs and accuracy of the Trained triangle showed an obvious learning effect after training and the effect was not transferred to the Untrained triangle.

#### EEG test phase

The behavioral analysis of the EEG phase focused on the accuracy and RTs by using a two‐tail paired‐samples *t*‐test. For accuracy, there were significant differences between searching for the Trained triangle and the Untrained triangle (Δ 0.50 ± 0.12, *t* (19) = 18.44, *p* < .001, Cohen's *d* = 4.23, 95% CI [0.45, 0.56]), and between searching for the Salient triangle and Untrained triangle (Δ 0.29 ± 0.17, *t* (19) = 7.74, *p* < .001, Cohen's *d* = 1.78, 95% CI [0.21, 0.37]). For RTs, there were significant differences between searching for the Trained triangle and Untrained triangle (Δ −13 ± 10 ms, *t* (19) = −5.77, *p* < .001, Cohen's *d* = 1.32, 95% CI [−0.17, −0.08]), and between searching for the Salient triangle and Untrained triangle (Δ −12 ± 8 ms, *t* (19) = −7.10, *p* < .001, Cohen's *d* = 1.63, 95% CI [−0.16, −0.09]). These results showed that the performance of searching for the Trained triangle and Salient triangle was better than searching for the Untrained triangle. We also analyzed the accuracy of searching for the Trained triangle and the Salient triangle when the Untrained triangle presented as a distractor, and the accuracy of searching for the Untrained triangle when the Trained triangle or Salient triangle presented as a distractor. The results showed that there were no significant differences between searching for the Trained triangle and Salient triangle (Δ −0.03 ± 0.26, *t* (19) = −0.60, *p* = .554, Cohen's *d* = 0.14, 95% CI [−0.15, 0.09]). There were significant differences between searching for the Untrained triangle when the Trained triangle or Salient triangle presented as distractor (Δ −0.19 ± 0.14, *t* (19) = −6.16, *p* < .001, Cohen's *d* = 1.41, 95% CI [−0.26, −0.13]).

### Electrophysiological markers

In the current study, we focused on four ERP components: N2pc, P_D_, N1, and P3. By comparing the amplitude and latency of these ERP components elicited by the target and distractor, we assessed the cognitive processes reflected by the salient triangle and the Trained triangle under the same task difficulty. Because the task of searching for the Untrained triangle was difficult and there were fewer trials for correct responses, the averaged ERPs in these analyses included all target‐present trials regardless of whether the response was correct or not.

The N2pc component reflected the shift of attention to a lateralized stimulus location (Luck & Hillyard, 1994a, 1994b), which was regarded as a neural marker to study attention capture effect (Hickey et al., 2006; Kiss et al., 2012; Töllner et al., 2012; Wykowska & Schubö, 2010). And P_D_ (distractor positivity) component reflected active suppression of distractor or stimuli unrelated to the task (Burra & Kerzel, 2014; Kiss et al., 2012; Sawaki & Luck, 2010; Sawaki et al., 2012). N2pc components, as well as P_D_ components, were obtained from the difference of the activity of PO7 and PO8 at the contralateral and ipsilateral posterior electrode sites. The onset latency was identified as the point of time when the voltage reaches 50% of the maximum negative or positive (Bachman et al., 2020). The average amplitudes for the N2pc and P_D_ components were calculated, respectively, within a 260–320‐ms and 450–520‐ms time window in the central RSVP task and a 240–340‐ms and 380–450‐ms time window in the visual search task.

The N1 component was a negative polarity wave which reflected the sensory processing evoked at the early stage (Bachman et al., 2020; Hopf et al., 2002; Vogel & Luck, 2000). We measured the N1 component through four parietal occipital electrode sites to explore the sensory processing in the early phase (P1, PZ, P2, and POZ). N1 components were derived from the average wave at the four electrode sites. The onset latency was identified as the point of time when the voltage reaches 50% of the maximum negative within the 160–220‐ms time window. We computed the average amplitudes for the N1 component within a 180–220‐ms time window.

The P3 component can reflect the outcome evaluation in the top‐down control process and can be used as an index of stimulus evaluation processes in cognitive processing (Bachman et al., 2020; Comerchero & Polich, 1999; Verleger et al., 2005). Analyses of P3 component focused on activity within the electrodes P1, P2, CZ, CP1, PZ, and CP2. P3 components were derived from the average wave at the seven electrode sites. The onset latency determined to be the point when the voltage reaches the 50% maximum positive within a 350–550‐ms time window. For the average amplitudes, the P3 were computed within a 450–550‐ms time window.

#### N2pc in both central RSVP and visual search tasks

**FIGURE 4:**
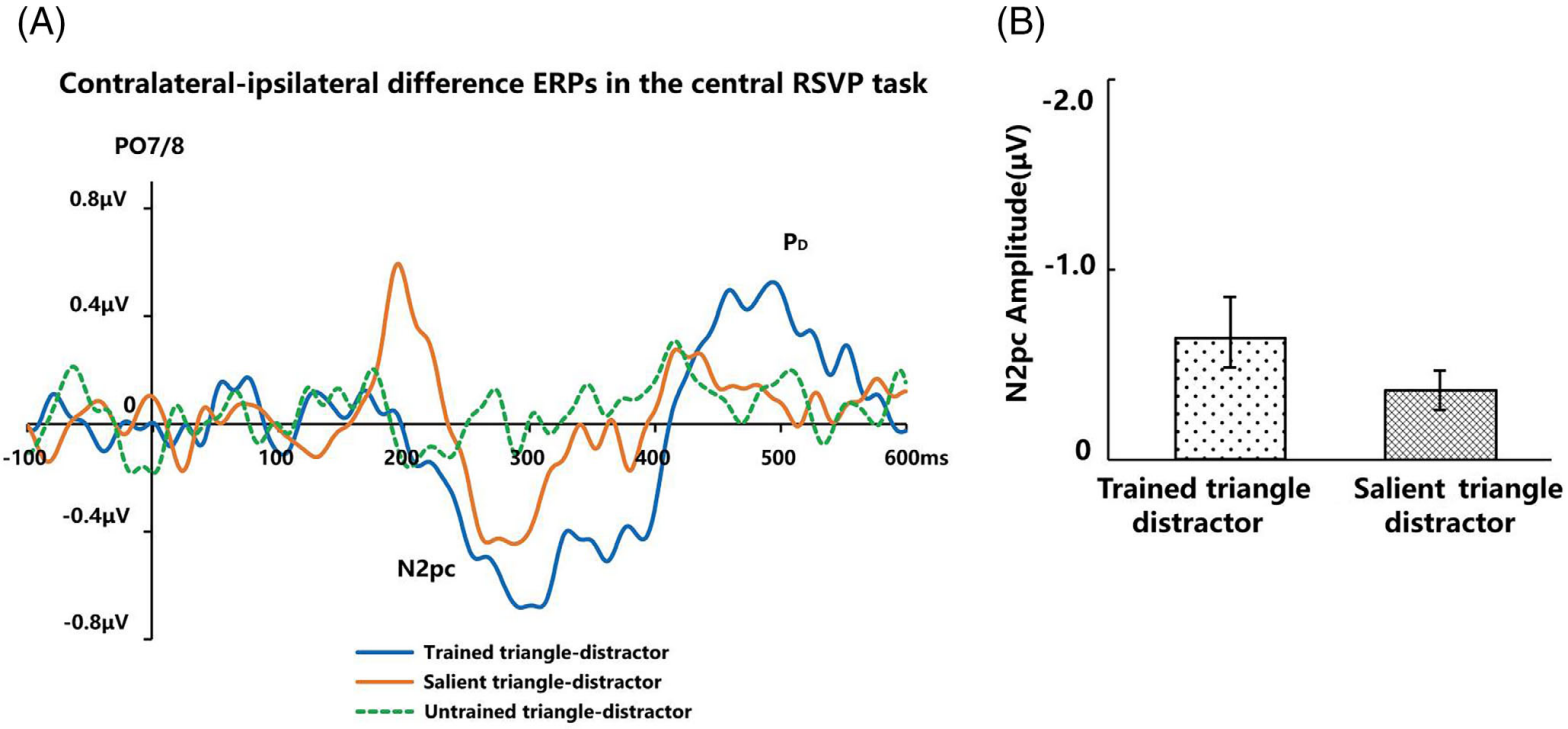
(A) Contralateral‐ipsilateral difference ERPs in the central RSVP task. (B) The results of N2pc amplitude in the central RSVP task.

For the RSVP task, among 18 subjects who participated in the analysis of the central RSVP task, 13 subjects received three type triangle pairs (see section EEG Test Phase) due to the adjustment of the subsequent experiment. The first five subjects received trained‐distractor triangle and salient‐distractor triangle pairs, and the subsequent 13 subjects added the untrained‐distractor triangle pairs due to the adjustment of the subsequent experiment (received trained‐distractor triangle, salient‐distractor triangle and untrained‐distractor triangle pairs). Therefore, we analyzed the N2pc components of 18 subjects and calculated the N2pc components of the 13 subjects who only received three triangle pairs (trained‐distractor triangle, salient‐distractor triangle, and untrained‐distractor triangle). We did not find significant difference in the significance of their results. Therefore, the subsequent results of the central RSVP task were for all 18 subjects. The N2pc components induced by the Trained triangle, Salient triangle and Untrained triangle are shown as Figure 4A. We found that the contralateral waveforms were more negative than the ipsilateral waveforms over the 260–320‐ms time window when the trained‐distractor triangle pair was presented (Δ −0.64 ± 0.76 μV, *t* (17) = −3.59, *p* = .002, Cohen's *d* = 0.87, 95% CI [−1.02, −0.26]) and salient‐distractor triangle (Δ −0.40 ± 0.45 μV, *t* (17) = −3.77, *p* = .002, Cohen's *d* = 0.91, 95% CI [−0.62, −0.17]). There was no significant difference between ipsilateral and contralateral wave when the untrained‐distractor triangle pair was presented (Δ 0.01 ± 0.32 μV, *t* (12) = 0.14, *p* = .892, Cohen's *d* = 0.04, *BF*_10_ = 0.28, 95% CI [−0.18, 0.20]). These results indicated that among the three type triangle pairs, the Trained triangle and Salient triangle could elicit an N2pc component, and the Untrained triangle could not elicit an N2pc component. We compared the difference in latency and amplitude (Figure 4B) of N2pc elicited by the Trained triangle and Salient triangle in the central RSVP task. A two‐tailed paired *t*‐test showed that there was no significant difference in latency (Δ −6.00 ± 30.34 ms, *t* (17) = −0.84, *p* = .413, Cohen's *d* = 0.20, *BF*_10_ = 0.33, 95% CI [−21.09, 9.09]) and amplitude (Δ −0.24 ± 0.81 ms, *t* (17) = −1.30, *p* = .210, Cohen's *d* = 0.32, *BF*_10_ = 0.50, 95% CI [−0.65, 0.15]). Although both the Salient triangle and the Trained triangle were able to capture attention automatically when presented as task‐irrelevant stimuli in the central RSVP task, there was no difference in latency and amplitude between the Trained triangle and Salient triangle.

**FIGURE 5:**
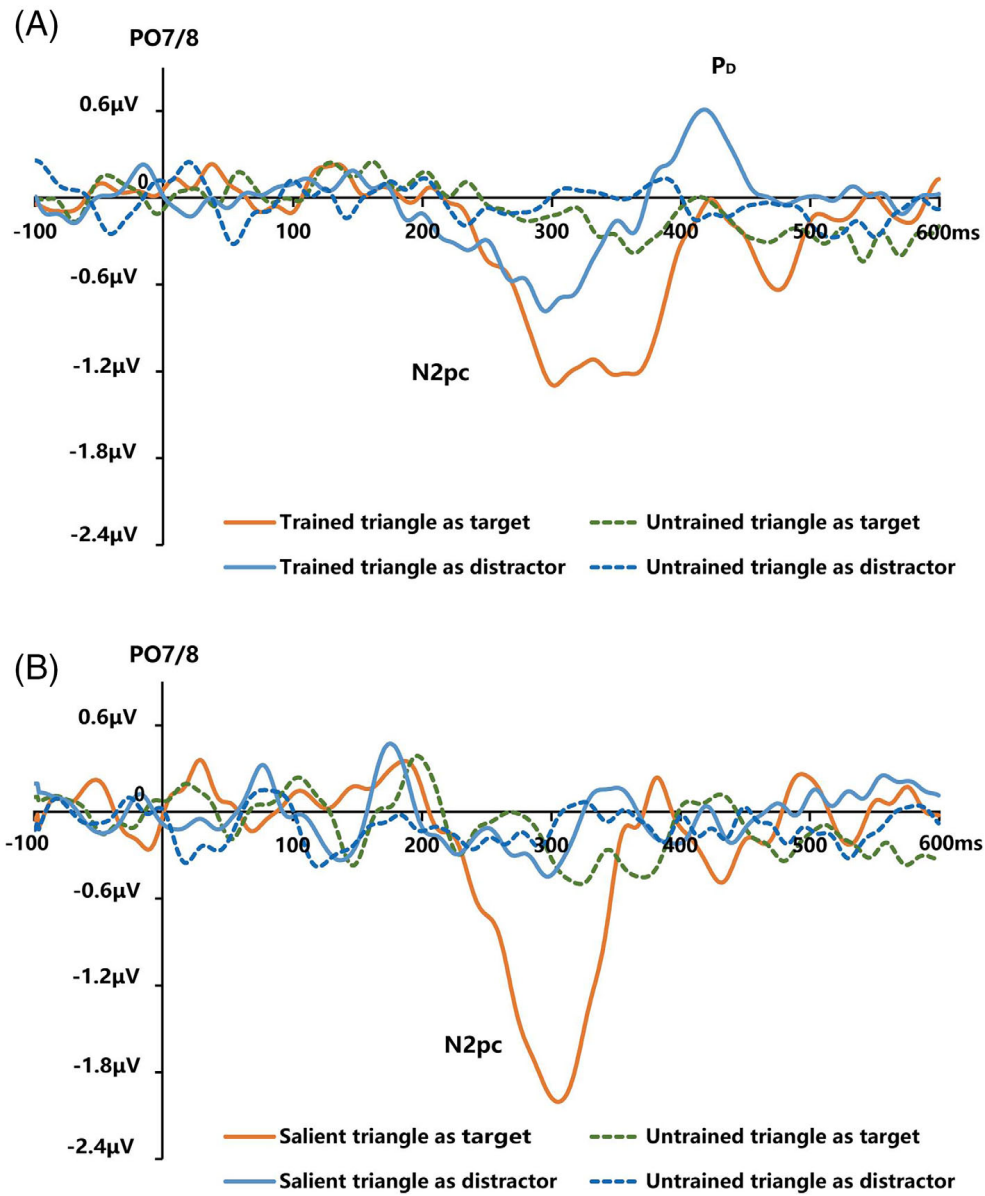
(A) Contralateral‐ipsilateral difference ERPs in the search tasks of searching for T_UnT and UnT_T. (B) Contralateral‐ipsilateral difference ERPs in the search tasks of searching for S_UnT and UnT_S.

**FIGURE 6:**
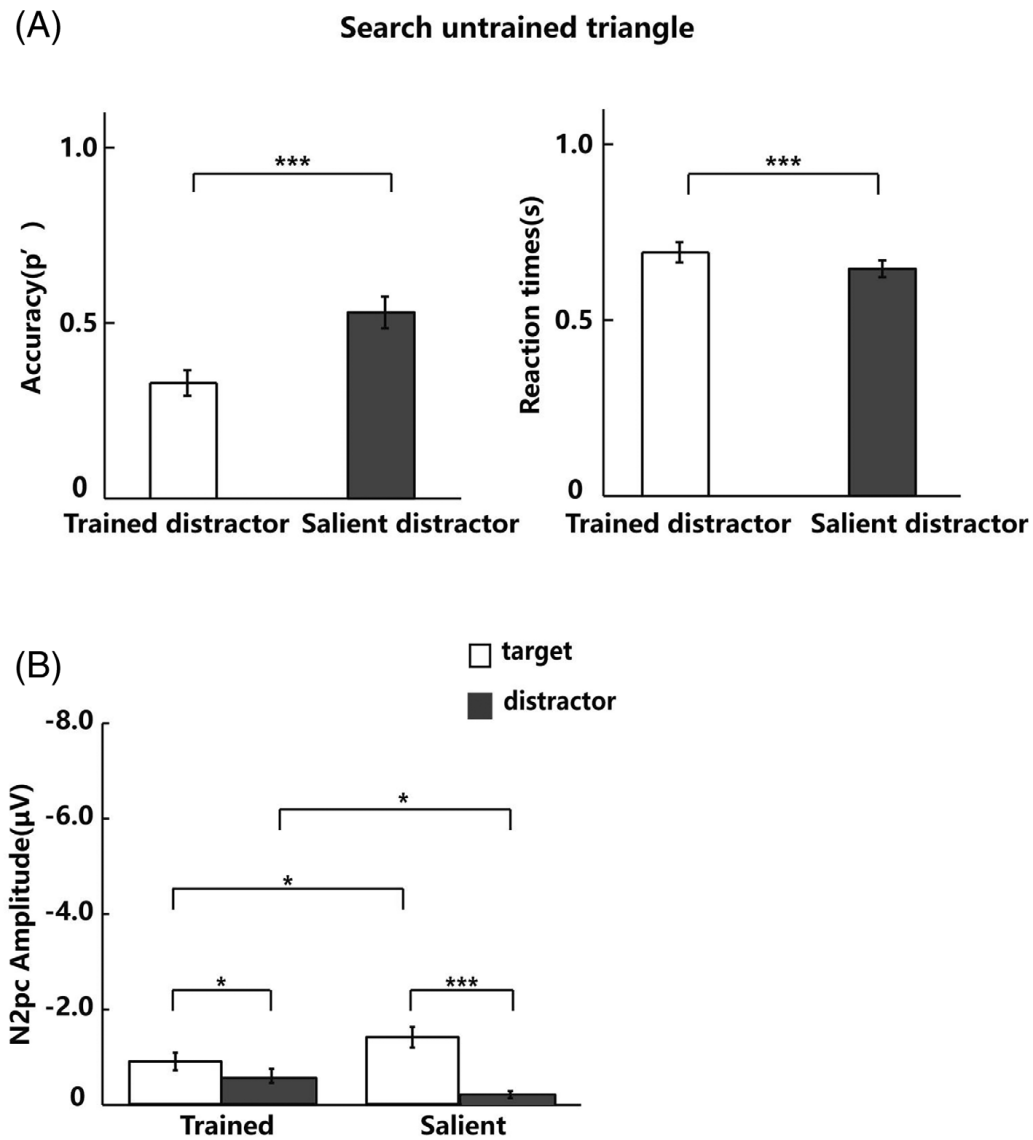
(A) The accuracy (p’) and RTs of searching Untrained triangle when Trained triangle and salient triangle served as distractor. (B) The results of N2pc amplitude in the visual search tasks.

For the visual search task, the N2pc components of four search tasks are illustrated as Figure 5A, B. In the search tasks of searching for T_UnT and UnT_T, the contralateral waveforms were more negative than the ipsilateral waveforms over the 240–340‐ms time window with the Trained triangle as target (Δ −0.90 ± 0.76 μV, *t* (19) = −5.25, *p* < .001, Cohen's *d* = 1.20, 95% CI [−1.25, −0.54]) and distractor (Δ −0.55 ± 0.60 μV, *t* (19) = −4.05, *p* = .001, Cohen's *d* = 0.93, 95% CI [−0.83, −0.26]), and there was no significant difference between ipsilateral and contralateral waves when the Untrained triangle was presented as the target (Δ −0.11 ± 0.26 μV, *t* (19) = −1.96, *p* = .065, Cohen's *d* = 0.45, *BF*_10_ = 1.12, 95% CI [−0.24, 0.01]) or distractor (Δ 0.04 ± 0.49 μV, *t* (19) = 0.32, *p* = .748, Cohen's *d* = 0.07, *BF*_10_ = 0.24, 95% CI [−0.20, 0.27]). In the search tasks of searching for S_UnT and UnT_S, the contralateral waveform was more negative relative to the ipsilateral waveform when the salient triangle was presented as the target (Δ −1.40 ± 0.90 μV, *t* (19) = 0.70, *p* < .001, Cohen's *d* = 1.61, 95% CI [−1.82, −0.98]) and distractor (Δ −0.19 ± 0.30 μV, *t* (19) = −2.84, *p* = .010, Cohen's *d* = −0.65, 95% CI [−0.34, −0.05]). However, we also found that the contralateral waveforms were more negative than the ipsilateral waveforms with the Untrained triangle as the target (Δ −0.22 ± 0.38 μV, *t* (19) = −2.62, *p* = .017, Cohen's *d* = 0.60, 95% CI [−0.40, −0.05]). There was no significant difference between the ipsilateral and contralateral waves when the Untrained triangle was presented as distractor (Δ −0.11 ± 0.35 μV, *t* (19) = −1.38, *p* = .184, Cohen's *d* = 0.32, *BF*_10_ = 0.53, 95% CI [−0.27, 0.06]). These results indicated that both the Trained triangle and Salient triangle elicited the N2pc component when they were presented as target and distractor, while the Untrained stimulus did not elicit N2pc in the task of searching for UnT_T. However, we found a much weaker N2pc when the Untrained triangle was presented as target (Salient triangle as one of the distractors). To explore why the Untrained target yielded different results when the distractor was the Trained triangle and Salient triangle, we compared the accuracy and RTs of searching for the Untrained triangle under the two search tasks (Figure 6A). The results of a two‐tailed paired *t*‐test showed a significant improvement in accuracy (Δ −0.20 ± 0.14, *t* (19) = −6.22, *p* < .001, Cohen's *d* = 1.43, 95% CI [−0.26, −0.13]) and RTs (Δ 5 ± 5 ms, *t* (19) = 4.43, *p* < .001, Cohen's *d* = 1.02, 95% CI [0.03, 0.08]). These results suggested that the Untrained triangle target might have been trained and elicited a weaker N2pc component when the Salient triangle was presented as distractor.

The previous analyses established whether the components were different from zero to determine the elicitation of N2pc, whereas the current analysis compares the conditions by ANOVA. We further compared the latency and amplitude of N2pc components induced by the Trained triangle and Salient triangle in the search tasks. For the latency of N2pc (the grand mean ± *SD*: 268.28 ms ± 2.55), the results of a repeated‐measure ANOVA with triangle type (Trained triangle vs. Salient triangle) and triangle condition (served as target vs. served as distractor) showed no significant effect on triangle condition (*F*(1, 19) = 0.15, *p* = .700, $\eta_{p}^{2}$ = .01), triangle type (*F*(1, 19) = 0.33, *p* = .572, $\eta_{p}^{2}$ = .02), or their interaction (*F*(1, 19) = 0.10, *p* = .755, $\eta_{p}^{2}$ = .01). For the amplitude of N2pc (Figure 6B), the results of a repeated‐measure ANOVA with triangle type (Trained triangle vs. Salient triangle) and triangle condition (served as target vs. served as distractor) showed significant effects on triangle condition (*F*(1, 19) = 42.17, *p* < .001, $\eta_{p}^{2}$ = .69) and their interaction (*F*(1, 19) = 11.04, *p* = .004, $\eta_{p}^{2}$ = .37). We found no significant main effect on triangle type (*F*(1, 19) = 0.33, *p* = .573, $\eta_{p}^{2}$ = .02). The results of further simple‐effects analysis (Bonferroni‐corrected) showed significant difference between the Trained triangle and the Salient triangle when they served as targets (Δ 0.50 ± 0.94 μV, *t* (19) = 2.41, *p* = .026, Cohen's *d* = 0.55, 95% CI [0.07, 0.95]) and distractors (Δ −0.35 ± 0.70 μV, *t* (19) = 2.25, *p* = .037, Cohen's *d* = 0.52, 95% CI [−0.68, −0.02]). For the Trained triangle and Salient triangle, the N2pc was significantly larger when they served as target than distractor (Trained triangle: Δ −0.35 ± 0.55 μV, *t* (19) = 2.84, *p* = .010, Cohen's *d* = 0.65, 95% CI [−0.60, −0.09]; Salient triangle: Δ −1.21 ± 0.97 μV, *t* (19) = −5.56, *p* < .001, Cohen's *d* = 1.28, 95% CI [−1.66, −0.75]). These results showed that there was no difference in latency between the Trained triangle and Salient triangle when both served as target and distractor. For the Trained triangle and Salient triangle, the amplitude was larger when they served as target than when they served as distractor, and the amplitude of the Salient triangle was larger than that of the Trained triangle when they served as target. However, the amplitude of the Trained triangle was larger than that of the Salient triangle when they served as distractor.

#### P_D_

In the central RSVP task, the contralateral waveforms of trained‐distractor triangle pair were more positive over the 450–520‐ms time window compared to the ipsilateral waveforms (Δ 0.47 ± 0.60 μV, *t* (17) = 3.34, *p* = .004, Cohen's *d* = 0.81, 95% CI [0.17, 0.77]). However, the contralateral waveforms of other triangle pairs were not significant compared with the ipsilateral waveforms (salient‐distractor triangle: Δ 0.10 ± 0.46 μV, *t* (17) = 0.94, *p* = .358, Cohen's *d* = 0.23, *BF*_10_ = 0.36, 95% CI [−0.13, 0.33]; untrained‐distractor triangle: Δ 0.11 ± 0.26 μV, *t* (12) = 1.52, *p* = .156, Cohen's *d* = 0.37, *BF*_10_ = 0.69, 95% CI [−0.05, 0.27]). The results showed that only the Trained triangle could induce a P_D_ component.

In the search tasks of searching for T_UnT and UnT_T, the contralateral waveform over the 380–450‐ms time window was more positive compared to the ipsilateral waveforms when the Trained triangle was distractor (Δ 0.39 ± 0.58 μV, *t* (19) = 3.01, *p* = .007, Cohen's *d* = 0.69, 95% CI [0.12, 0.66]). However, there was no significant difference between ipsilateral and contralateral waves when the Untrained triangle was presented as target (Δ −0.11 ± 0.48 μV, *t* (19) = −1.05, *p* = .306, Cohen's *d* = 0.24, *BF*_10_ = 0.38, 95% CI [−0.34, 0.11]) or distractor (Δ −0.11 ± 0.55 μV, *t* (19) = −0.91, *p* = .375, Cohen's *d* = 0.21, *BF*_10_ = 0.34, 95% CI [−0.37, 0.15]), as well as the Trained triangle as the targets (Δ −0.29 ± 1.01 μV, *t* (19) = −1.30, *p* = .210, Cohen's *d* = 0.30, *BF*_10_ = 0.48, 95% CI [−0.77, 0.18]). In the search tasks of searching for S_UnT and UnT_S, we did not observe more positive waveforms on the contralateral than the ipsilateral when the salient triangle was presented as the target (Δ −0.21 ± 0.62 μV, *t* (19) = −1.54, *p* = .139, Cohen's *d* = 0.35, *BF*_10_ = 0.64, 95% CI [−0.51, 0.08]) or the distractor (Δ −0.13 ± 0.28 μV, *t* (19) = −2.14, *p* = .051, Cohen's *d* = 0.49, 95% CI [−0.26, −0.00]). Similarly, the ipsilateral and contralateral waves were not significantly different whether the Untrained triangle served as target (Δ −0.01 ± 0.63 μV, *t* (19) = −0.04, *p* = .966, Cohen's *d* = 0.01, *BF*_10_ = 0.23, 95% CI [−0.30, 0.29]) or distractor (Δ −0.11 ± 0.37 μV, *t* (19) = −1.35, *p* = .192, Cohen's *d* = 0.31, *BF*_10_ = 0.52, 95% CI [−0.29, 0.06]). These results suggested that only the Trained triangle serving as distractor could elicit P_D_ components.

#### N1

**FIGURE 7:**
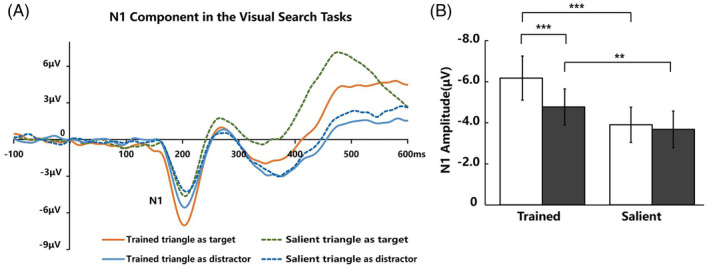
(A)The waveforms of N1 component in the visual search tasks. (B) The results of N1 amplitude in the visual search tasks ***p < .001. **p < .01.

Figure 7A illustrates the N1 waveforms elicited by the Trained triangle and Salient triangle when they presented as target and distractor. We analyzed the latency over the 160–220‐ms time window and amplitude of N1 over the 180–220‐ms time window elicited by the Trained triangle and salient triangle. For the latency of N1, the results of a repeated‐measure ANOVA with triangle type (Trained triangle vs. Salient triangle) and triangle condition (served as target vs. served as distractor) showed significant effect on triangle type (*F*(1, 19) = 9.65, *p* = .006, $\eta_{p}^{2}$ = .33). We did not find significant effect on triangle condition (*F*(1, 19) = 1.74, *p* = .202, $\eta_{p}^{2}$ = .08), or their interaction (*F*(1, 19) = 2.55, *p* = .127, $\eta_{p}^{2}$ = .12). These results suggested that the Trained triangle had a shorter latency than the salient triangle both serving as target and distractor.

For the amplitude of N1 (Figure 7B), the results of a repeated‐measure ANOVA with triangle type (Trained triangle vs. Salient triangle) and triangle condition (served as target vs. served as distractor) showed significant effect on triangle condition (*F*(1, 19) = 14.39, *p* = .001, $\eta_{p}^{2}$ = .43), triangle type (*F*(1, 19) = 32.74, *p* < .001, $\eta_{p}^{2}$ = .63) and their interaction (*F*(1, 19) = 8.00, *p* = .011, $\eta_{p}^{2}$ = .30). The results of further simple‐effects analysis (Bonferroni‐corrected) revealed that the waveforms elicited by the Trained triangle were more negative than the salient triangle in both triangle conditions (serving as target: Δ −2.22 ± 1.66 μV, *t* (19) = −5.98, *p* < .001, Cohen's *d* = 1.37, 95% CI [−3.00, −1.14]; serving as distractor: Δ −1.14 ± 1.47 μV, *t* (19) = −3.46, *p* = .003, Cohen's *d* = 0.79, 95% CI [−1.82, −0.45]). The amplitude of N1 elicited by the Trained triangle was significantly larger when they were presented as target than distractor (Δ −1.34 ± 1.38 μV, *t* (19) = −4.35, *p* < .001, Cohen's *d* = 1.00, 95% CI [−1.99, −0.70]). However, we did not find significant difference when the Salient triangle presented as target and distractor (Δ −0.26 ± 1.16 μV, *t* (19) = −0.99, *p* = .335, Cohen's *d* = 0.23, *BF*_10_ = 0.36, 95% CI [−0.80, 0.29]). In summary, the Trained triangle had a shorter latency and larger amplitude than the Salient triangle both serving as target or distractor.

#### P3

**FIGURE 8:**
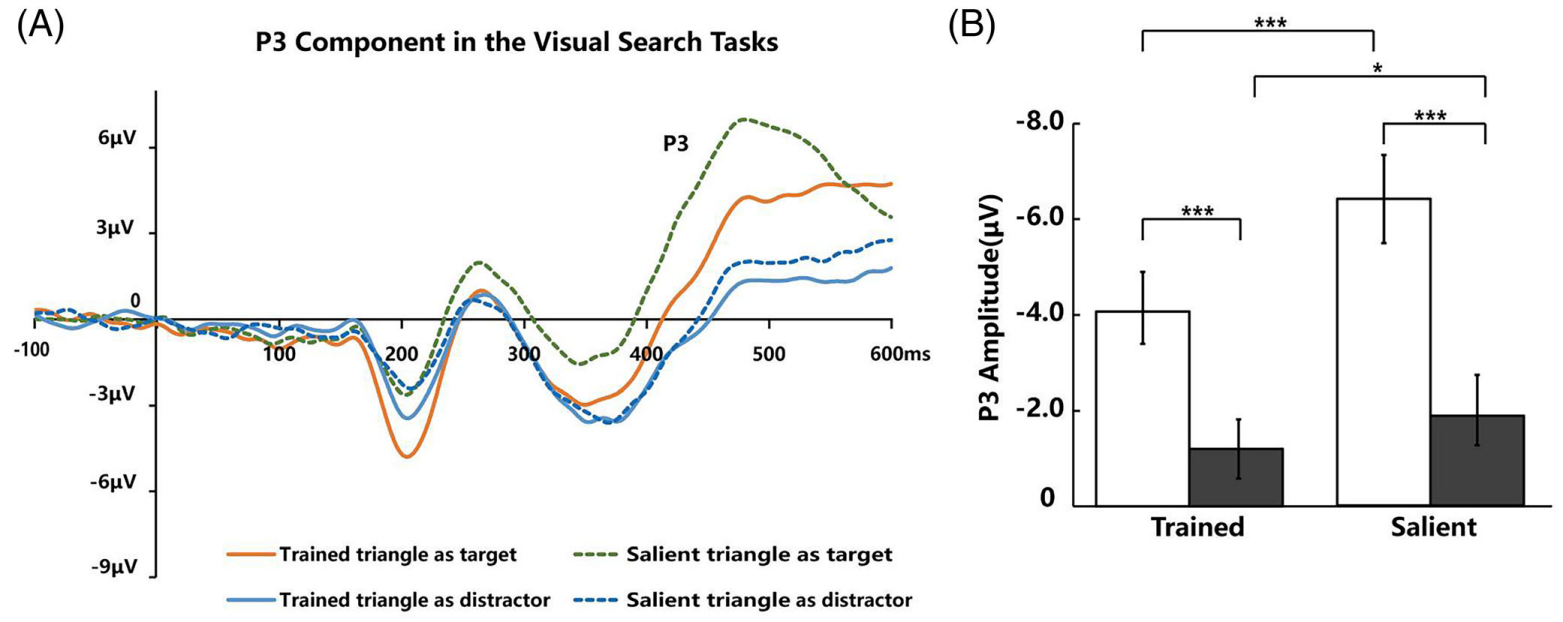
(A) The waveforms of P3 component in the visual search tasks. (B) The results of P3 amplitude in the visual search tasks.

The P3 waveforms elicited by the Trained triangle and Salient triangle when they served as targets and distractors are shown as Figure 8A. We analyzed the latency over the 350–550‐ms time window and amplitude of P3 over the 450–550‐ms time window elicited by the Trained triangle and Salient triangle. Repeated‐measure ANOVA with the triangle type (Trained triangle vs. Salient triangle) and triangle condition (served as target vs. served as distractor) as factors were conducted on latency and amplitude of P3. For latency, we did not find significant effect on triangle type (*F*(1, 19) = 3.07, *p* = .096, $\eta_{p}^{2}$ = .14), triangle condition (*F*(1, 19) = 1.40, *p* = .252, $\eta_{p}^{2}$ = .07) or their interaction (*F*(1, 19) = 2.05, *p* = .17, $\eta_{p}^{2}$ = .10).

For amplitude (Figure 8B), there were significant effects on triangle type (*F*(1, 19) = 25.74, *p* < .001, $\eta_{p}^{2}$ = .58), triangle condition (*F*(1, 19) = 83.89, *p* < .001, $\eta_{p}^{2}$ = .82), and their interaction (*F*(1, 19) = 15.40, *p* = .001, $\eta_{p}^{2}$ = .45). Simple‐effects analysis (Bonferroni‐corrected) revealed that the waveforms elicited by the salient triangle were more negative than the Trained triangle both serving as target (Δ −2.34 ± 1.93 μV, *t* (19) = −5.41, *p* < .001, Cohen's *d* = 1.24, 95% CI [−3.24, −1.43]) and distractor (Δ −0.70 ± 1.26 μV, *t* (19) = −2.47, *p* = .023, Cohen's *d* = 0.57, 95% CI [−1.29, −0.11]). For the Trained triangle and Salient triangle, the P3 was significantly larger when the they served as target than distractor (Trained triangle: Δ 2.90 ± 1.83 μV, *t* (19) = 7.07, *p* < .001, Cohen's *d* = 1.62, 95% CI [2.04, 3.76]; Salient triangle: Δ 4.54 ± 2.23 μV, *t* (19) = 9.10, *p* < .001, Cohen's *d* = 2.09, 95% CI [3.49, 5.58]).

## DISCUSSION

In the present study, we investigated nonsalient trained stimuli and physically salient stimuli through similar or different neural mechanisms, including examining what such differences might be. The results of the test phase showed that the performance of Trained triangles could be improved in the accuracy and RTs after training. These results suggested that the training was effective, and the training effect did not transfer to Untrained triangles. The Trained triangles could induce N2pc components and capture attention when presented as targets and distractors, and as peripheral irrelevant stimuli in the central RSVP task. Different from previous studies, we also considered the difference between salient and nonsalient trained stimuli. The results showed that the Salient triangles could elicit N2pc components both presented as targets or distractors. They could automatically capture attention as well as Trained triangles. These results were consistent with the previous findings on salient stimuli (Burra & Kerzel, 2014; Kiss et al., 2012). Since all peripheral stimuli were task‐irrelevant in the RSVP task, the elicitation of N2pc by nonsalient Trained triangles and physically Salient triangles suggested that they resulted in involuntary attentional capture. In fact, we cannot completely exclude top‐down attentional control. Because the nonsalient Trained triangle was trained over 3 days, this history guidance meant that attention capture was not purely bottom‐up. Apparently, the old attentional template (nonsalient Trained triangle) still affected the deployment despite an unrelated new attentional set (the blue letter). Moreover, there are two functions of attention suppression that we mentioned before. If the N2pc component disappeared without subsequent P_D_ component, attention suppression may serve to prevent a shift of attention; if the N2pc component was followed by the P_D_ component, it may terminate a shift of attention. In current study, we observed that the P_D_ component followed the N2pc when Trained triangles served as distractors, which reflected that the shift of attention is terminated by the active suppression mechanism. After training, the capture of distractors remained present and the reduced RTs indicated faster disengagement (Theeuwes, 2010; Wang et al., 2019). In the present study, we matched the difficulty of physically Salient triangles and Trained triangles between the two stimuli in visual search. For N2pc latency, we found no significant difference between Trained triangles and Salient triangles in either the central RSVP task or visual search task. These results suggested that there was no significant difference between the Trained triangles and Salient triangles on the speed of the attentional‐shifting. For amplitude, the N2pc amplitude of Trained triangles was significantly larger than Salient triangles when they served as distractors and was weaker when they were presented as targets. This suggested that physically salient targets specifically created a larger attentional selection response when they served as targets, but nonsalient Trained triangles had a stronger ability to capture attention when they were presented as distractors. These results provided clear neural evidence that the two stimuli modulated attentional selection via different neural mechanisms. P_D_ components could be elicited when Trained triangles were presented as distractors in the visual search tasks and peripheral irrelevant stimuli in the central RSVP task. Those results are consistent with the study of Hu et al. (2018). In their study, the T shapes of four possible orientations (Up, Down, Left, or Right) were presented as nonsalient stimuli. However, in the study of Qu et al. (2017), nonsalient trained triangles only elicited the N2pc component, but not the P_D_ component when presented as distractors. Hu et al. (2018) speculated that the trained targets could not elicit the P_D_ component because of the difficulty of the task. In the current study, the task difficulty was decreased by reducing the set size (mean accuracy *p*' in Qu et al.'s study: 0.35 vs. current study: 0.85). The present results showed that nonsalient Trained triangles could elicit the P_D_ component while adjusting for task difficulty using a similar paradigm of Qu et al. (2017). These results indicated that nonsalient Trained triangles as a distractor did not elicit P_D_, which might be due to the difficulty of the task. Specifically, we did not observe P_D_ components when Salient triangles served as distractors in the visual search task and peripheral irrelevant stimuli in the central RSVP task, which were different from previous studies (Gaspar & McDonald, 2014; Gaspelin & Luck, 2018). In fact, it was found that the P_D_ component induced by salient stimuli when serving as distractors may be related to individual differences. Individuals with high capacity could successfully suppress salient distractors, while individuals with low capacity could not suppress salient distractors (Gaspar et al., 2016). In the current study, the same subjects completed all the tasks. Therefore, these results may be due to the different neural mechanisms of the Trained triangles and Salient triangles. According to Hu et al. (2018), it is crucial to consider that the P_D_ component is elicited only when the distractor's detectability is relatively high. Therefore, the absence of P_D_ component for salient stimuli at the same task difficulty may be because the salient stimuli may not require suppression due to insufficient salience.

We also explored early sensory processing by comparing the differences between the latency and amplitude of the N1 component elicited by Trained triangles and Salient triangles. Previous studies have found that the N1 component is related to the early sensory processing of attentional selection in visual search (Bachman et al., 2020; Hopf et al., 2002; Vogel & Luck, 2000). The results showed that the Trained triangles had a shorter latency and larger amplitude than the Salient triangles under the same task difficulty. The shorter latency indicated that the Trained triangles had an earlier sensory processing. Compared to the Salient triangles, the greater amplitude of the Trained triangles seemed to reflect the greater visual attention. Nevertheless, the amplitude of P3 component elicited by Salient triangles was stronger than that of Trained triangles, which indicated that the top‐down controlled process of outcome evaluation for Salient triangles was stronger. Thus, those results suggested Trained triangles and physically Salient triangles through the different mechanisms in early sensory processing of the stimulus input and evaluation processes in cognitive processing.

Finally, although the results in the search tasks of searching for S_UnT and UnT_S were consistent with the previous study, we did find that the Untrained triangle targets induced a weaker N2pc when salient triangles served as distractors. It seemed that we used the same Untrained triangles in the test phase and EEG test phase with a total of 1,104 trials, which led to Untrained triangles being trained. In this case, it may be the cause of capturing attention when it served as targets in the later stage (the last search task). To demonstrate this, we compared the accuracy and RTs of searching for Untrained triangles under the two search tasks. The improvement in performance of searching for Untrained triangles when Salient triangles were presented as distractors provided the evidence supporting our hypothesis. However, the current study still cannot rule out the effect of difficulty on the difference in results. Because we focused on aligning the difficulty of nonsalient trained stimuli and the salient stimuli as the target rather than as the distractor in the matching phase, the difficulty of the untrained stimulus as the target condition was not sufficiently controlled. This raised the possibility that the results observed in both cases could still be affected by the confounding factor of difficulty. Therefore, it is necessary to exclude the influence of difficulty when comparing the result difference between the salient stimulus and the trained stimulus presented as distractors in future studies.

## CONCLUSION

Generally, the current study provided clear neural evidence for whether the physically salient triangle and Trained triangle operate through the different neural mechanisms in visual search. In the early sensory processing, the Trained triangle had an earlier sensory processing and greater visual attention. For attentional selection, the physically salient triangles created a larger attentional selection response when they served as targets and the nonsalient trained triangles had a stronger ability to capture attention when they were presented as distractors. In the outcome evaluation process, the top‐down controlled process of outcome evaluation for the salient triangle was stronger.

## CONFLICT OF INTEREST STATEMENT

All authors certify that they have no affiliations with or involvement in any organization or entity with any financial interest or non‐financial interest in the subject matter or materials discussed in this manuscript.

## ETHICS STATEMENT

The studies involving human participants were reviewed, written informed consent was obtained from the participants and the study was approved by the Psychology and Education Research Ethics Committee of Minnan Normal University (Ethics approval number: No. 2022‐06‐01).
